# Different Chondrogenic Potential among Human Induced Pluripotent Stem Cells from Diverse Origin Primary Cells

**DOI:** 10.1155/2018/9432616

**Published:** 2018-01-21

**Authors:** Yeri Alice Rim, Yoojun Nam, Narae Park, Hyerin Jung, Yeonsue Jang, Jennifer Lee, Ji Hyeon Ju

**Affiliations:** ^1^Catholic iPSC Research Center, College of Medicine, The Catholic University of Korea, Seoul 137-701, Republic of Korea; ^2^Division of Rheumatology, Department of Internal Medicine, Seoul St. Mary's Hospital, Institute of Medical Science, College of Medicine, The Catholic University of Korea, Seoul 137-701, Republic of Korea

## Abstract

Scientists have tried to reprogram various origins of primary cells into human induced pluripotent stem cells (hiPSCs). Every somatic cell can theoretically become a hiPSC and give rise to targeted cells of the human body. However, there have been debates on the controversy about the differentiation propensity according to the origin of primary cells. We reprogrammed hiPSCs from four different types of primary cells such as dermal fibroblasts (DF, *n* = 3), peripheral blood mononuclear cells (PBMC, *n* = 3), cord blood mononuclear cells (CBMC, *n* = 3), and osteoarthritis fibroblast-like synoviocytes (OAFLS, *n* = 3). Established hiPSCs were differentiated into chondrogenic pellets. All told, cartilage-specific markers tended to express more by the order of CBMC > DF > PBMC > FLS. Origin of primary cells may influence the reprogramming and differentiation thereafter. In the context of chondrogenic propensity, CBMC-derived hiPSCs can be a fairly good candidate cell source for cartilage regeneration. The differentiation of hiPSCs into chondrocytes may help develop “cartilage in a dish” in the future. Also, the ideal cell source of hiPSC for chondrogenesis may contribute to future application as well.

## 1. Introduction

Reprogramming mature somatic cells into human induced pluripotent stem cells (hiPSCs) opened a new strategy in tissue engineering and regenerative medicine. The delivery of transcription factors (i.e., OCT4, SOX2, KLF4, and c-Myc) transits somatic cells into a state similar to embryonic stem cells. The pluripotency and unlimited proliferation makes hiPSCs an ideal cell source for the production of transplantable regenerative cell sources.

In the early years of hiPSC generation, skin fibroblasts were usually used for reprogramming. The first hiPSCs generated by Yamanaka himself was generated from human skin dermal fibroblasts (DF). DFs may be convenient to cultivate in vitro; however, it can be obtained only through an invasive punch biopsy procedure. Somatic cells that are easy to obtain from an individual were required as a substitute. Today, hiPSCs are generated from various cell sources such as blood cells, keratinocytes, urine cells, and more [1–3].

Previous reports suggest that the original primary cell source of hiPSCs can subsequently affect the in vitro differentiation ability. Some report that the differentiation ability is biasing the cells towards the tissue of origin [4–8]. Epigenetic memory such as DNA methylation is thought to be responsible for the different differentiation capacity [9, 10].

Through our previous studies, we successfully generated hiPSCs from peripheral blood mononuclear cells (PBMCs) and cord blood mononuclear cells (CBMCs) [11, 12]. We also generated hiPSCs using fibroblast-like synoviocytes (FLSs) isolated from the synovium in the knee joint of osteoarthritis (OA) patients [13, 14]. CBMC-derived hiPSCs were generated with CBMCs with homozygous human leukocyte antigen (HLA) types for future use in regenerative medicine. Using the CBMC-derived hiPSCs, we confirmed the potential in chondrogenic differentiation by generating chondrogenic pellets that are about 1-2 mm in size [15, 16]. After 21 days of differentiation using CBMC-hiPSCs in chondrogenic differentiation medium containing human BMP2 and TGF*β*3, the chondrogenic pellets showed increased expression of chondrogenic markers (i.e., ACAN, COL2A1, COMP, and SOX9). Extracellular matrix (ECM) proteins were positively detected in the CBMC-hiPSC-derived chondrogenic pellets and the quality was comparable with that generated using actual mesenchymal stem cells (MSCs), which is a generally used cell source for in vitro chondrogenesis.

It is well known that the adult articular cartilage lacks natural healing ability [17–20]. Chondrogenesis using hiPSCs was attempted for several years with hiPSCs generated from various origin cells (i.e., adipose-derived stem cells, fibroblasts, and articular chondrocytes) [15, 21–25]. All cell sources successfully went through chondrogenesis. However, to our knowledge, the capacity of chondrogenic differentiation between hiPSCs generated from different origins was not confirmed. This study aims to compare the chondrogenic efficacy of hiPSCs generated from PBMCs, DFs, FLSs, and CBMCs.

## 2. Materials and Methods

### 2.1. Dermal Fibroblast and Fibroblast-Like Synoviocyte Isolation and Maintenance

Skin samples were extracted by skin punch biopsy procedures. Synovium samples were received by arthroscopic synovectomy or total knee replacement surgery. The delivered skin and synovium was chopped and homogenized. Chopped tissues were resuspended in Dulbecco's modified Eagle's medium (DMEM, Gibco, Carlsbad, CA, USA) containing with 0.01% collagenase. Tissues were digested with collagenase for 4 hours at 37% with vigorous shaking. Cells were washed and resuspended in DMEM supplemented with 20% fetal bovine serum (FBS, Gibco) and 1% penicillin/streptomycin solution (Gibco). Cells were cultured until more than 80% confluence was achieved.

### 2.2. Peripheral Blood and Cord Blood Mononuclear Cell Isolation and Maintenance

Blood was delivered in heparin tubes. Fresh blood was diluted with phosphate buffered saline (PBS) and placed onto Ficoll-Paque reagent. The samples were centrifuged for 30 minutes at 850 ×g. Isolated PBMCs were transferred to a new tube and washed with PBS. Cell pellet was resuspended in StemSpan media (STEMCELL Technologies, Vancouver, Canada) containing CC110 cytokine (STEMCELL Technologies) for expansion. Cells were incubated for 5 days before reprogramming. Fresh media was added if needed.

### 2.3. hiPSC Generation Using Dermal Fibroblasts and Synoviocytes

Cells were washed and treated with trypsin/EDTA. Cells were counted and 3 × 10^5^ cells per well was obtained. Cells were then resuspended in 20% DMEM and seeded into a 6-well plate. Yamanaka factors were delivered by sendai virus (Invitrogen, Carlsbad, CA, USA). The next day, prealiquoted sendai virus was treated to the cells and incubated in 5% CO_2_, 37°C for 48 hours. After 48 hours, virus-containing media were removed and changed every other day for 6 days. On day 7, media were changed to hiPSC media. Cells were cultured until colonies appeared. Media were changed daily after they were transited to E8 media.

### 2.4. iPSC Induction Using Peripheral Mononuclear Blood Cells and Cord Blood Mononuclear Cells

Reprogramming and characterization was performed as previously described [12]. Cells transfected with sendai virus containing Yamanaka factors were maintained in a vitronectin-coated dish (Thermo Fisher Scientific, Waltham, MA, USA), and media were changed daily with fresh E8 medium (STEMCELL Technologies) before use. Cells were maintained in vitronectin-coated dishes. Media were changed daily.

### 2.5. Chondrogenic Differentiation Using Pellet Culture

The protocol was followed by the procedure shown in our previous studies [15, 16]. A 1 : 1 mixture of TeSR-E8 and AggreWell media (STEMCELL Technologies) was added to 2 × 10^6^ hiPS cells for EB generation. EBs were maintained in E8 media for 3 days and then in E7 media for additional 3 days. EBs were harvested and resuspended in outgrowth (OG) cell induction media consisted of DMEM, 20% FBS, and 10% penicillin/streptomycin. EBs were counted and 50–70 EB per cm^2^ was seeded onto a gelatin-coated dish. OG cells were induced from the attached EBs for 3 days at 5% CO_2_, 37°C. Next, cells were detached and the remaining EB clumps were removed using a 40 *μ*m cell strainer (BD Technologies, Franklin Lakes, NJ, USA). Single OG cells were harvested and plated onto a new gelatin-coated dish (1–5 × 10^4^ cell per cm^2^). Cells were used up to passage 5. OG cells were counted and 3 × 10^5^ cells per pellet were prepared. Cells were harvested in a 15 mL conical tube and media were changed into chondrogenic differentiation media (CDM; DMEM supplemented with 20% knockout serum replacement, 1x nonessential amino acids, 1 mM L-glutamine, 1% sodium pyruvate, 1% ITS+ Premix, 10^−7^ M dexamethasone, 50 mM ascorbic acid, and 40 *μ*g/mL L-proline) supplemented with 10 ng/mL recombinant human bone morphogenetic protein 2 (BMP2), and transforming growth factor beta 3 (TGF*β*3). Cells resuspended in CDM were centrifuged at 750 ×g for 5 minutes. Generated pellets were maintained for 21 days and media were changed every 3 days.

### 2.6. Polymerase Chain Reaction

Chondrogenic pellets or cells were harvested and stored at −80°C. Samples were snap frozen with liquid nitrogen and ground using a pestle. Ground samples were incubated with TRIzol (Thermo Fisher Scientific), and mRNA was extracted according to the manufacturer's instructions. Using RevertAid™ First Strand cDNA Synthesis kit (Thermo Fisher Scientific), 2 *μ*g of the extracted total RNA was used to synthesize cDNAs. Real-time PCR reactions included 2 *μ*L of diluted cDNA (1 : 10 dilution). Real-time PCR was carried out using LightCycler® 480 Instrument II (Roche, Basel, Switzerland). The geometric mean of GAPDH was used as an internal control to normalize the results.

The detection of viral vectors was followed by the manufacturer's instruction produced in the reprogramming sendai virus kit (Invitrogen). Reverse transcription reaction was performed with the synthesized cDNAs. Integration of viral vectors was confirmed using four primers; SeV, KOS, KLF4, and c-Myc. The primer sequences are provided in Table 1.

### 2.7. Histological Analysis of Pellets

Chondrogenic pellets were washed with PBS. Pellets were fixed in 4% paraformaldehyde for 2 hours at room temperature (RT). Dehydration was performed with increasing sequential ethanol solutions. Additional clearing was done with sequential ethanol-xylene mixtures and pellets were infiltrated with paraffin overnight. Paraffin blocks were fixed and 7 *μ*m sections were obtained using a microtome. Before staining the sections, slides were placed in a 60°C oven for at least 10 minutes. Slides were immediately deparaffinized using xylene. Slides were rehydrated with decreasing sequential ethanol series and were rinsed with running tap water for 1 minute each. Toluidine blue staining was done by incubating the hydrated slides in 0.04% toluidine blue (Sigma Aldrich, St. Louis, Missouri, USA) solution for 10 minutes. Slides were washed in running tap water and dried for 10 minutes until complete dryness. After the staining process, slides were dehydrated with an increasing sequential ethanol series. Ethanol was cleared with 2 cycles of 100% xylene and slides were mounted with VectaMount™ Permanent Mounting Medium (Vector Laboratories, Burlingame, CA, USA).

### 2.8. Immunohistochemistry

Slides were placed in a 60°C oven for 10 minutes and deparaffinized with 2 cycles of xylene. Slides were rehydrated and were incubated in boiling citrate buffer (Sigma Aldrich) for antigen unmasking. After cooling the unmasked slides, endogenous peroxidase activity was blocked by treating the slides with 3% hydrogen peroxide (Sigma Aldrich). Slides were washed and blocked with tris-buffered saline (TBS) containing 1% bovine serum albumin (BSA). Primary antibodies were diluted in blocking solution (collagen type II, 1/100; collagen type I, 1/200; collagen type X, 1: 250, Abcam). Slides were incubated with diluted primary antibody at 37°C for 1 hour. Slides were washed with TBS containing 0.1% tween-20. Secondary antibodies (1/200; Vector Laboratories) diluted in blocking buffer were treated for 40 minutes at RT. After washing out the secondary antibody, slides were treated with ABC reagent drops (Vector Laboratories) for 30 minutes. DAB solution (Vector Laboratories) was followed and incubated for 5 minutes. Slides were washed and counterstained with Mayer's hematoxylin (Sigma Aldrich) for 1 minute. Slides were dehydrated and cleared. Slides were mounted and staining was confirmed under a bright-field microscope.

### 2.9. Statistical Analysis

Comparison of each of cell type-derived chondrogenic pellets and hiPSCs was analyzed by Kruskal-Wallis one-way ANOVA followed by Dunn's multiple comparison test. Analysis was done using the software GraphPad Prism 5 (^∗^ *P* < 0.05; ^∗∗^ *P* < 0.01; and ^∗∗∗^ *P* < 0.001 for statistically significant differences).

## 3. Results

### 3.1. Generation of hiPSCs from a Different Origin

Human iPSCs were generated from DFs, PBMCs, FLSs and CBMCs. The information of each cell line is shown in Table 2. Somatic cells were obtained from three individual donors per cell type. Some of the PBMCs and DFs were obtained from the identical donor. FLSs were isolated from the joint of three osteoarthritis patient's synovium. Three hiPSC clones were generated from each hiPSC and three chondrogenic pellets were analyzed individually from each cell line.

**Figure 1:**
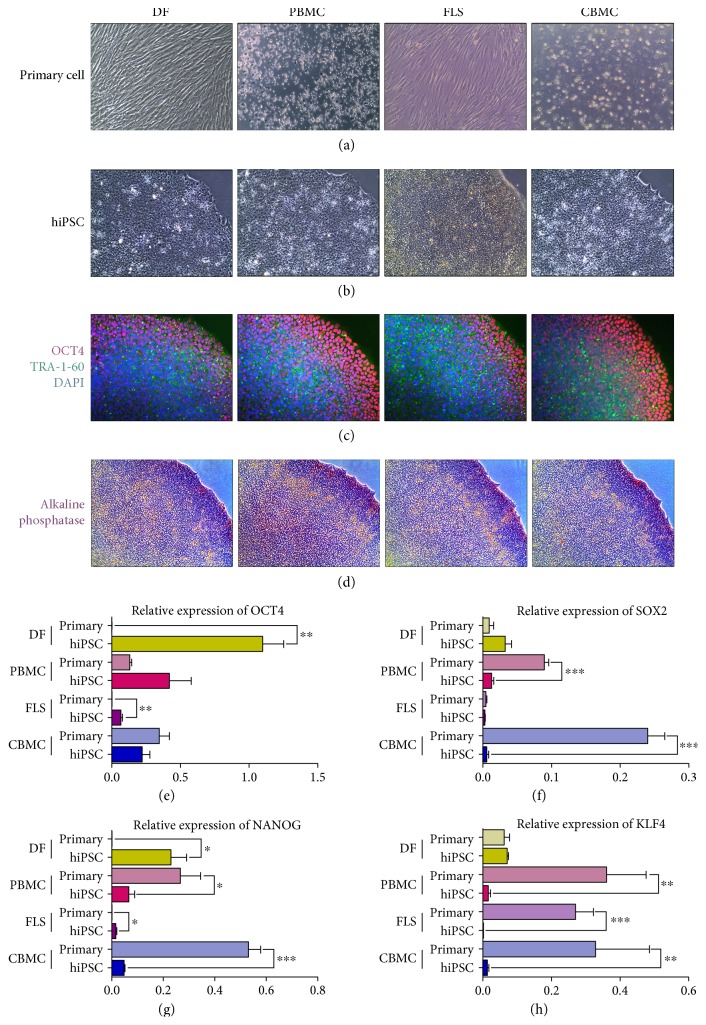
Characterization of DF-, PBMC-, FLS-, and CBMC-hiPSCs. (a) The morphology of each primary cell type: dermal fibroblast (DF), peripheral blood mononuclear cell (PBMC), fibroblast-like synoviocyte (FLS), and cord blood mononuclear cell (CBMC). (b) The morphology of the generated hiPSCs. (c) Immunohistology image of the hiPSC colonies stained against OCT4 and TRA-1-60. (d) Image of colonies stained against alkaline phosphatase (AS). (e) Relative expression of pluripotent markers in each cell line and primary cells (^∗^ *P* < 0.05; ^∗∗^ *P* < 0.01; and ^∗∗∗^ *P* < 0.001).

Primary cells were isolated from skin tissue, blood, synovium, and cord blood (Figure 1(a)). Primary cells were transduced with sendai viral vectors containing Yamanaka reprogramming factors. After several passaging and purification, reprogrammed cells exhibited a colony with compact morphology (Figure 1(b)). Pluripotency of the generated hiPSCs was confirmed with several markers. Colonies of each cell line showed positive expression of OCT4 and TRA-1-60 (Figure 1(c)). The colonies were also positively stained against alkaline phosphatase (AP) (Figure 1(d)). By real-time PCR, the relative expression of OCT4, SOX2, NANOG, and KLF4 was assessed in hiPSCs generated from different somatic cells (Figures 1(e)–1(h)). Interestingly, the expression of pluripotent markers was higher in PBMC and CBMC primary cells. Also, KLF4 expression was comparably high in FLSs as well. All of the pluripotent markers were expressed highest in DF-hiPSCs. The tendency of each pluripotent marker showed an identical pattern of expression. The silencing of integration of reprogramming viral vectors was confirmed in the hiPS cell lines (Figure S1). We have confirmed that the generated hiPSCs were viral vector free. Taken all together, we successfully reprogrammed nonintegrated hiPSCs from DFs, PBMCs, FLSs, and CBMCs with confirmed pluripotency.

### 3.2. Chondrogenic Pellet Generation Using hiPSCs from a Different Origin

**Figure 2:**
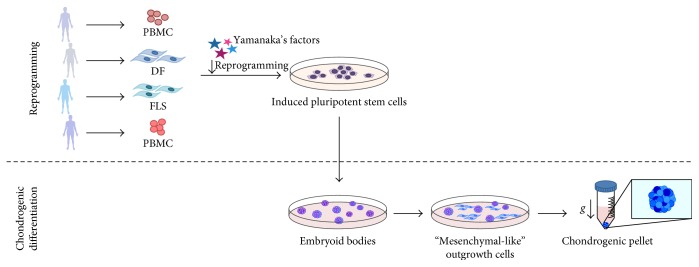
Scheme of experiment. Human iPSCs generated from different originated somatic cells were condensed into embryoid bodies (EB). EBs were attached to gelatin-coated dishes and outgrowth (OG) cells were induced. Using the OG cells, chondrogenic pellets were generated with centrifugal force and human growth factors.

In our previous studies, we confirmed the chondrogenic differentiation ability of CBMC-hiPSCs [15, 16]. The protocol we used is simply described in Figure 2. Roughly, embryoid bodies (EBs) were generated using hiPSCs. EBs were cultivated for about 1 week and attached onto a gelatin-coated plate for outgrowth (OG) cell induction, which are cells that sprout out from the bottom of EBs. OG cells are known to have the similar characteristics of MSCs [26]. Koyama et al. referred them as “mesenchymal progenitor cells.” Sprouted OG cells were then cultivated and used for chondrogenic differentiation. After 21 days of differentiation towards the chondrogenic lineage, pellets showed similar characteristics to that of cartilage (i.e., lacuna).

**Figure 3:**
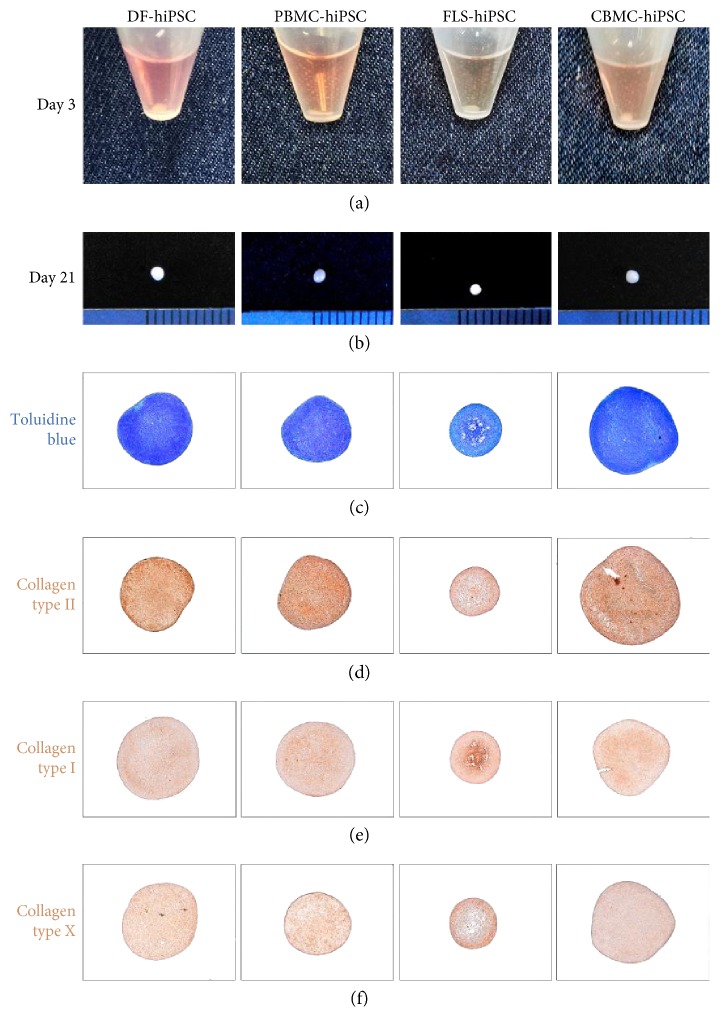
Chondrogenic pellets generated from hiPSC-derived from different originated somatic cells. (a) Morphology of outgrowth cells 3 days after differentiation. (b) Chondrogenic pellets after 21 days of chondrogenic differentiation. (c) Toluidine staining and (d) collagen type II immunohistology images of the chondrogenic pellets generated from each cell source-hiPSCs. (e) Collagen type I and (f) collagen type X staining images of chondrogenic pellets.

On day 3 of differentiation, OG cells derived from each hiPSC were condensed into a pellet (Figure 3(a)). OG cells derived from DF-hiPSCs and CBMC-hiPSCs condensed faster than the other two, and PBMC-hiPSCs took the longest time to form a pellet. After 21 days of differentiation, chondrogenic pellets were harvested (Figure 3(b)). CBMC- and DF-derived chondrogenic pellets had the largest size, indicating larger amounts of accumulated ECM proteins (Figure S2). On the other hand, FLS-derived pellets had the smallest size; however, there was no significant difference when the size were measured. ECM proteins were detected by toluidine blue staining (Figure 3(c)). Pellets generated from CBMC-hiPSCs showed high ECM accumulation through staining. PBMC- and DF-hiPSC-derived pellets also showed highly accumulated ECM proteins. However, FLS-hiPSC-derived pellets showed less accumulation. Cell death in the inner area was also detected, despite the small pellet size. The results of staining against collagen type II showed a similar tendency (Figure 3(d)). The staining in FLS-hiPSC-derived chondrogenic pellets showed hallow areas in the inner area. The other three cell types showed similar expression of collagen type II. The expression of collagen type I (Figure 3(e)) and type X (Figure 3(f)) was confirmed as well. The expression of both collagen types was highly detected in the inner area of the FLS-derived chondrogenic pellet. The other cell type-derived pellets had similar levels of collagen types I and X.

### 3.3. Chondrogenic Gene Expression in Chondrogenic Pellets Originating from Different Cell Source-Derived iPSCs

**Figure 4:**
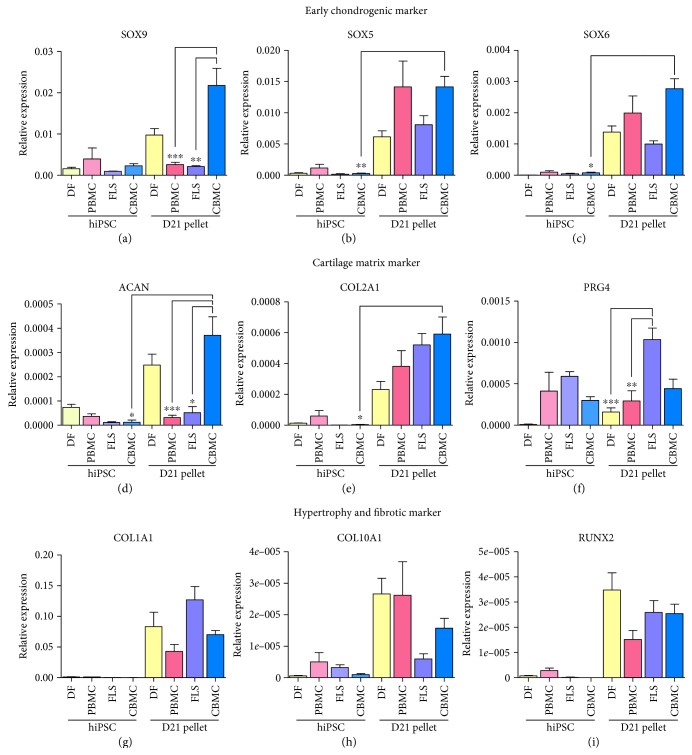
Gene expression in chondrogenic pellets. The expression of transcriptional activators or early chondrogenesis markers (a) SOX9, (b) SOX5, and (c) SOX6 was confirmed. The expression of matrix proteins, (d) aggrecan (ACAN), (e) collagen type II (COL2A1), and lubricin (PRG4) was confirmed. Also, hypertrophy or fibrotic markers such as (g) collagen type I (COL1A1), (h) collagen type X (COL10A1), and (i) RUNX2 was confirmed as well. To confirm the gene expression in each cell type, *n* = 9–18 pellets were used in this experiment (^∗^ *P* < 0.05; ^∗∗^ *P* < 0.01; and ^∗∗∗^ *P* < 0.001).

The gene expression of chondrogenic pellets generated from CBMC-derived hiPSCs (*n* = 27) was examined and compared with pellets derived from DF- (*n* = 27), PBMC- (*n* = 27), and FLS-hiPSC- (*n* = 18) derived pellets. SOX9 is generally known as the transcriptional activator that is crucial for chondrogenesis. The expression of SOX9 was highest in CBMC-hiPSCs (Figure 4(a)). The expression was significantly higher than that of PBMC-hiPSCs or FLS-hiPSCs. SOX5 and SOX6 are closely related to the DNA-binding proteins that enhance the function of SOX9 [27]. The expression of SOX5 and 6 was also confirmed in the chondrogenic pellets. The expression of SOX5 in CBMC-hiPSC-derived pellets was significantly higher than DF- and FLS-hiPSC-derived chondrogenic pellets (Figure 4(b)). PBMC-hiPSC-derived pellets had similarly high expression of SOX5. This was also shown in SOX6 as well (Figure 4(c)). Aggrecan (ACAN) and collagen type II (COL2A1) are the major proteoglycan proteins in the articular cartilage that provides the hydrated gel structure. The expression of ACAN correlated to that of SOX9 (Figure 4(d)). ACAN expression was significantly higher in CBMC-hiPSC-derived chondrogenic pellets. Similar to SOX9, the expression pattern was not significant between DF-hiPSC-derived pellets and CBMC-hiPSC-derived pellets. Also, CBMC-hiPSC-derived pellets were the only type that showed significantly increased marker expression compared to its primary cell source. ACAN expression in PBMC- and FLS-hiPSC-derived pellets was relatively lower than the other two. In the case of COL2A1 expression, however, DF-hiPSC-derived pellets had the lowest expression (Figure 4(e)). Identical to ACAN expression, CBMC-hiPSC-derived pellets were the only type that showed significantly increased COL2A1 expression compared to its primary cell source. Lubricin (PRG4) is a proteoglycan that acts as lubricant in the joint. Interestingly, the expression of lubricin was significantly high in FLS-hiPSC-derived pellets (Figure 4(f)). On the other hand, hiPSCs derived from PBMCs, FLSs, and CBMCs all had a relatively high expression of PRG4. Collagen type I (COL1A1) is a general marker for fibrocartilage, which is a less lubricated form of cartilage. As it is also a fibrotic marker, the expression was higher in FLS-hiPSC-derived pellets (Figure 4(g)). COL1A1 expression was high in both DF- and FLS-hiPSC-derived pellets. PBMC-hiPSC-derived pellets had the lowest expression of COL1A1 and FLS-hiPSC-derived pellets showed the highest expression. Yet, despite these differences, the results lacked significance. Collagen type X (COL10A1) is a marker for hypertrophy. The expression of COL10A1 was mostly all lower than other markers (Figure 4(h)). Hypertrophy can lead to osteogenecity, and RUNX2 is another marker indicating early calcification or osteogenesis of cartilage. RUNX2 expression was the highest in DF-hiPSC-derived chondrogenic pellets (Figure 4(i)). The expression was significantly lower in PBMC-hiPSC-derived pellets compared to CBMC-hiPSC-derived pellets. However, the expression of RUNX2 was relatively lower than all the other markers in every cell type-derived chondrogenic pellets and it also lacks significance between all samples. The levels of pluripotent markers were measured in the generated chondrogenic pellets (Figure S3). The expression levels of OCT4, SOX2, and NANOG were all decreased in the generated pellets. The most important hiPSC marker, NANOG, was most significantly decreased in the CBMC-derived chondrogenic pellets. However, KLF4 expression was increased in all cases. Through these results, CBMC-hiPSC-derived pellets had a fairly superior quality than the other three cell sources. Yet, there was not much morphological difference between CBMC-, PBMC-, and DF-derived cell lines. The expression of pluripotent markers was also confirmed in chondrogenic pellets (Figure S3). The expression of hiPSC markers (i.e., OCT4, SOX2, and NANOG) was downregulated in differentiated chondrogenic pellets. However, the expression of KLF4 was increased in the chondrogenic pellets.

### 3.4. Gene Expression in Pellets Generated from hiPSCs Reprogrammed from Cells Sharing the Same Genetic Identity

**Figure 5:**
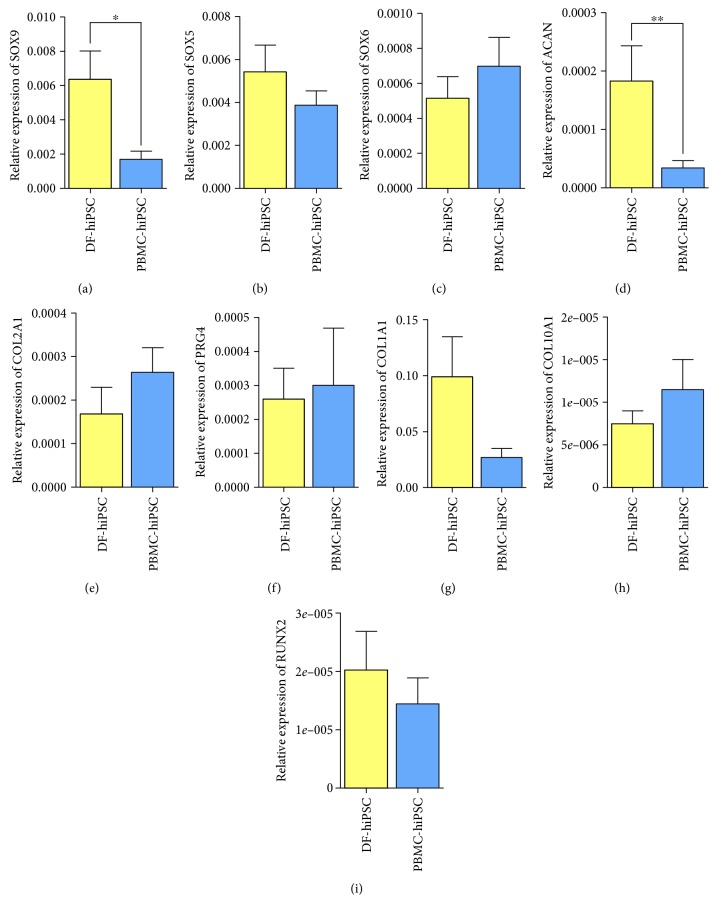
The expression of DF- and PBMC-hiPSC-derived chondrogenic pellets generated from the same donor. The expression of early transcription markers: (a) SOX9, (b) SOX5, and (c) SOX6. The expression of proteoglycan matrix proteins: (d) ACAN, (e) COL2A1, and (f) PRG4. The expression fibrotic and hypertrophic markers: (g) COL1A1, (h) COL10A1, and (i) RUNX2 (^∗^ *P* < 0.05; ^∗∗^ *P* < 0.01).

Two of each of PBMC- and DF-hiPSCs were obtained from the same donor. To compare the chondrogenic potential from different cell sources (DFs and PBMCs) obtained from the same donor, we profiled the chondrogenic and other marker expression. Similar to the results of Figure 4(a), the expression of SOX9 was significantly higher in DF-hiPSC-derived pellets even in genetically identical conditions (Figure 5(a)). The expression of SOX5 turned out to be the opposite of the earlier data (Figure 4(b)); however, it was shown that the difference was not significant (Figure 5(b)). The expression of SOX6 was higher in PBMC-hiPSC-derived pellets as predicted; however, there was no significance as well (Figure 5(c)). The expression of ACAN was significantly higher in DF-hiPSC-derived chondrogenic pellets (Figure 5(d)). This was identical to the results shown in Figure 4(d). COL2A1 and PRG4 expression also showed a similar pattern as shown in Figure 4 (Figures 5(e) and 5(f)). The expression of COL1A1 was higher in DF-hiPSC-derived pellets (Figure 5(g)). COL10A1 expression was higher in PBMC-hiPSC-derived pellets (Figure 5(h)); however, the expression of RUNX2 was lower than DF-hiPSC-derived pellets. Taken all together, DF-hiPSC-derived pellets showed higher expression of the early transcription marker SOX9 and proteoglycan gene ACAN. Most of the markers had no significant difference in the chondrogenic pellets generated from PBMC- and DF-hiPSCs sharing the same genetic identity.

## 4. Discussion

In this present study, the chondrogenic differentiation potential of hiPSCs generated from different donor cell sources was investigated. Chondrogenic pellets were generated using several cell sources: CBMC, PBMC, DF, and FLSs. Between these samples, two of each of PBMCs and DFs were genetically identical. FLSs were obtained from an osteoarthritis patient's synovial tissue. The somatic cell source seemed to affect the chondrogenecity of hiPSCs. Our group previously showed the chondrogenic differentiation potential of CBMC-derived hiPSCs [15]. Compared to other cell sources, CBMC-hiPSC-derived pellets showed relatively higher expression of early and late (proteoglycan) markers of chondrogenesis. This data suggest that CBMC-hiPSCs can be used as a comparable cell source for in vitro chondrogenesis or cartilage regeneration.

In vitro chondrogenesis is an important issue, since the original tissue has limited capacity to regenerate or heal. Successful generation of hyaline cartilage in tissue engineering and in vitro cultivation systems can lead to tremendous therapeutic benefits in the related fields. Early in vitro chondrogenesis was done using MSCs or adipocyte-derived stem cells [20, 28]. These two adult stem cells are also actually used in current clinics along with autologous chondrocytes for the regeneration of the damaged cartilage [29–31]. Despite their high chondrogenic potential, the low coverage of the cells was the limit of these cell sources. Also, long in vitro cultures to proliferate these cells have the possibility of hypertrophy and dedifferentiation of cells [32, 33]. The low cellularity in elderly or patients may also impair the therapeutic effects of these cells [34]. Embryonic stem cells (ESCs) were mentioned for cartilage regeneration as an alternative; however, the ethical issues related to the embryo destructions are hard to overcome in several institutes. The emergence of hiPSCs was a breakthrough because of these reasons. The unlimited proliferation by self-renewal and high pluripotency was similar to that of ESCs, and the ethical issues were removed [35]. The spontaneous differentiation of hiPSCs also resulted in cartilage-like tissues, indicating the potential ability of hiPSCs in chondrogenesis.

The generation of hiPSCs can be theoretically done using any adult somatic cells. Various cell types (i.e., dermal fibroblast, blood cell, urine cell, and keratinocyte) were shown to reprogram successfully into hiPSCs [1, 3, 36–42]. Because of these reasons, hiPSCs are now widely used in the fields of disease modeling and drug screening that can replace the use of diseased animal models [43]. It is also the cutting edge material for tissue regeneration. However, the cell source used in reprogramming is now on the issue, since it might affect the outcome of reprogramming and regeneration. We generated hiPSCs from DFs, PBMCs, FLSs, and CBMCs. The generated hiPSCs had similar protein expression levels of pluripotent markers (i.e., OCT4 and TRA-1-60) (Figure 1), yet the genetic expression rather differed. The genetic expression levels of pluripotent markers such as OCT4, SOX2, NANOG, and KLF4 were highly expressed in DF-derived hiPSCs. PBMC-derived hiPSCs had the second highest expression and FLS-derived hiPSCs had the lowest. Interestingly, the expression of pluripotent markers was higher in the primary blood cells (PBMCs and CBMCs). This is thought to be caused by the effect of the maintenance media of suspension cells which can increase the hematopoietic stem cell population. Also, the expression of KLF4 in FLS-derived hiPSCs was highly expressed (Figure 1(h)). This fact was already confirmed in our previous report [13]. This finding is still being investigated in our other projects; however, a recent study reported that KLF4 is an inflammatory regulator in FLSs via IL-6 signaling [44].

While improved understandings and several protocols were developed in chondrogenic differentiation using hiPSCs, the selection of the donor somatic cell source used in reprogramming has become an important issue [25]. In this study, genetically identical PBMC- and DF-hiPSC were differentiated into chondrogenic pellets (Figure 5). Our results suggested that the two cell sources eventually had no significant difference in chondrogenic potential under the same genetic circumstances. The expression of SOX9 and ACAN correlated and were the only markers that shown significant difference between the two differently originated hiPSCs. Both SOX9 and ACAN were significantly higher in DF-hiPSC-derived pellets. We concluded that some somatic cell sources might not have different outcomes in chondrogenesis. Yet, further examination and more cell lines that share the same genetic profiles to confirm chondrogenesis might be required before we assert these results.

The expression of pluripotent markers were examined in the chondrogenic pellets (Figure S3). The expression of OCT4, SOX2, and NANOG was decreased in chondrogenic pellets compared to hiPSCs. Interestingly, KLF4 expression was increased in all chondrogenic pellets generated from different cell types. This data can suggest that KLF4 might play an important role in chondrogenesis. The relation between KLF4 and chondrogenesis is not fully understood at this point; however, Outani and colleagues previously reported induction of chondrogenesis using KLF4 with other factors [45, 46]. Chondrogenic cells were directly induced from mouse dermal fibroblasts by transducing only SOX9, c-Myc, and KLF4. This might explain the reason of the increased levels of KLF4 in cells undergoing chondrogenesis. Also, based on the fact that FLS-hiPSCs had high expression of KLF4 (Figure 1(h)), it might be a characteristic of the pathology of OA. Previous reports such as the one reported by Liu et al. suggest that KLF4 inhibit the expression of IL-1*β*, which is the main cytokine that is thought to be closely related to OA. Further research on this subject may suggest another clue about the epigenetic memory of hiPSCs.

The inheritance of the initial epigenomes and characteristics of the primary cell source to the hiPSCs are mentioned as “epigenetic memory” [47]. This phenomenon makes hiPSC a reasonable source for disease modeling. It is thought that using patient-specific hiPSCs may reflect the pathological characteristics of the diseased somatic cell or individual. We also differentiated OA FLS-derived hiPSCs into chondrogenic pellets in this study. The pellets generated using FLS-derived hiPSCs showed low viability in the inner region, even though it had a relatively small size (Figure S2). The gene expression of OA FLS-hiPSC-derived chondrogenic pellets was also interesting. The expression of SOX9 was significantly lower in FLS-hiPSC-derived chondrogenic pellets (Figure 4(a)). The expression of the other two transcription factors, SOX5 and SOX6, was also low, compared to that of CBMC-hiPSC-derived chondrogenic pellets (Figures 4(b) and 4(c)). The expression of ACAN was also significantly lower than that DF- and CBMC-hiPSC-derived pellets. While the expression of COL2A1 and ACAN are usually known to have decreased expression in OA, however, COL2A1 expression was not that low than any other cell sources except CBMC-hiPSCs. Lubricin, or PRG4, is a protein in the cartilage ECM and synovial fluid that participates in the lubrication of the joint articular cartilage [48]. Interestingly, the expression of PRG4 was significantly higher than the other cell types. The expression of PRG4 was predicted to be decreased in OA; however, increased expression of PRG4 was also confirmed in OA patient human anterior cruciate ligament-MSCs [48]. Correlating with the previous reports, the high expression of PRG4 in OA might be an interesting subject to investigate. As other studies suggest that mechanical motions promote the expression of PRG4 in the articular cartilage, the high expression of PRG4 might be related to OA caused by external trauma or mechanical forces [49].

## 5. Conclusions

In conclusion, our CBMC-hiPSCs generated chondrogenic pellets with fair quality. However, no significant morphological difference was shown in normal hiPSC-derived pellets. With additional well-known benefits of the CBMCs (i.e., cell banking systems, stored HLA-typing information, and homozygous HLA types), the use of HLA-homozygous CBMC-hiPSCs have several advantages for future use of hiPSCs in clinics and tissue transplantation [50]. Our study suggests CBMC-hiPSCs as an ideal source for future application in cartilage regeneration. Also, we suggest hiPSCs generated from different somatic cells with different genetic and epigenetic backgrounds as a tool to assess and understand chondrogenesis and the cartilage in vitro.

## Figures and Tables

**Table 1:** Primers used for real-time PCR against pluripotent markers and chondrogenic markers.

Description	Target name	REFSEQ_ID	Direction	Primer sequence	Size
Pluripotency marker	OCT4	NM_203289.5	Forward	GGGAAATGGGAGGGGTGCAAAAGAGG	151
			Reverse	TTGCGTGAGTGTGGATGGGATTGGTG	
	KLF4	NM_004235.4	Forward	TTCCCATCTCAAGGCACAC	158
			Reverse	GGTCGCATTTTTGGCACT	
	SOX2	NM_003106.3	Forward	GGGAAATGGGAGGGGTGCAAAAGAGG	151
			Reverse	TTGCGTGAGTGTGGATGGGATTGGTG	
	NANOG	NM_024865.2	Forward	AAAGGCAAACAACCCACT	270
			Reverse	GCTATTCTTCGGCCAGTT	

Viral vector detection marker	SeV		Forward	GGATCA CTA GGTGATATCGAGC	181
			Reverse	ACCAGACAAGAGTTTAAGAGATATGTATC	
	KOS		Forward	ATGCACCGCTACGACGTGAGCGC	528
			Reverse	ACCTTGACAATCCTGATGTGG	
	Klf4		Forward	TTCCTGCATGCCAGAGGAGCCC	410
			Reverse	AATGTATCGAAGGTGCTCAA	
	c-Myc		Forward	TAACTGACTAGCAGGCTTGTCG	532
			Reverse	TCCACATACAGTCCTGGATGATGATG	

Chondrogenic marker	SOX9	NM_000346	Forward	GACTTCCGCGACGTGGAC	99
			Reverse	GTTGGGCGGCAGGTACTG	
	SOX5	NM_001261415.2	Forward	CAGCCAGAGTTAGCACAATAGG	104
			Reverse	CTGTTGTTCCCGTCGGAGTT	
	SOX6	NM_033326.3	Forward	GGATGCAATGACCCAGGATTT	141
			Reverse	TGAATGGTACTGACAAGTGTTGG	
	ACAN	NM_001135.3	Forward	TCGAGGACAGCGAGGCC	85
			Reverse	TCGAGGGTGTAGCGTGTAGAGA	
	COL2A1	NM_001844	Forward	GGCAATAGCAGGTTCACGTACA	79
			Reverse	CGATAACAGTCTTGCCCCACTTA	
	CHAD	NM_001267	Forward	GATCCCCAAGGTGTCAGAGAAG	66
			Reverse	GCCAGCACCGGGAAGTT	
	PRG4	NM_005807.4	Forward	AAAGTCAGCACATCTCCCAAG	199
			Reverse	GTGTCTCTTTAGCGGAAGTAGTC	
	COL1A1	NM_000088.3	Forward	TCTGCGACAACGGCAAGGTG	146
			Reverse	GACGCCGGTGGTTTCTTGGT	
	COL10A1	NM_000493.3	Forward	CAGGCATAAAAGGCCCAC	108
			Reverse	GTGGACCAGGAGTACCTTGC	
	RUNX2	NM_001024630	Forward	CCAGATGGGACTGTGGTTACTG	65
			Reverse	TTCCGGAGCTCAGCAGAATAA	

Housekeeping gene	GAPDH	NM_002046.5	Forward	ACCCACTCCTCCACCTTTGA	101
			Reverse	CTGTTGCTGTAGCCAAATTCGT	

**Table 2:** Information of generated hiPSCs.

hiPSC type	No	Sex	Cell type	Description
DF-hiPSC	1	F	Dermal fibroblast	Donor number 1
	2	M		Donor number 2
	3	M		Donor number 3

PBMC-hiPSC	1	F	Peripheral blood Mononuclear cell	Donor number 1
	2	M		Donor number 2
	3	F		Donor number 4

FLS-hiPSC	1	F	Synovial fibroblast	OA patient number 1
	2	F		OA patient number 2
	3	F		OA patient number 3

CBMC-hiPSC	1	F	Cord blood mononuclear cell	Donor number 5
	2	M		Donor number 6
	3	M		Donor number 7

## Supplementary Materials

Supplementary 1 Figure S1: Confirmation of vector-free hiPSCs. The elimination of viral vectors was confirmed in generated hiPSCs. The expression was confirmed in clone #1 of each hiPS cell line.

Supplementary 2 Figure S2: Size of chondrogenic pellets generated from hiPSCs. Chondrogenic pellets generated from CBMC-derived hiPSCs had the largest size.

Supplementary 3 Figure S3: The expression of pluripotent markers (OCT4, SOX2, NANOG, and KLF4) in day 21 chondrogenic pellets. The expression was compared to that of hiPSCs.
